# Comorbidity Patterns in Patients with Chronic Diseases in General Practice

**DOI:** 10.1371/journal.pone.0032141

**Published:** 2012-02-16

**Authors:** Luis García-Olmos, Carlos H. Salvador, Ángel Alberquilla, David Lora, Montserrat Carmona, Pilar García-Sagredo, Mario Pascual, Adolfo Muñoz, José Luis Monteagudo, Fernando García-López

**Affiliations:** 1 Multiprofessional Education Unit for Family and Community Care (Southwest), Madrid, Spain; 2 Telemedicine and e-Health Unit, Carlos III Institute of Health, Madrid, Spain; 3 Multiprofessional Education Unit for Family and Community Care (Center), Madrid, Spain; 4 Clinical Epidemiology Unit, 12 de Octubre University Teaching Hospital, 12 de Octubre Hospital Biomedical Research Institute (imas12), Madrid, Spain; 5 Bioengineering and Telemedicine Unit, Puerta de Hierro University Teaching Hospital, Madrid, Spain; 6 Clinical Epidemiology Unit, Puerta de Hierro University Teaching Hospital, Madrid, Spain; University of Michigan, United States of America

## Abstract

**Introduction:**

Healthcare management is oriented toward single diseases, yet multimorbidity is nevertheless the rule and there is a tendency for certain diseases to occur in clusters. This study sought to identify comorbidity patterns in patients with chronic diseases, by reference to number of comorbidities, age and sex, in a population receiving medical care from 129 general practitioners in Spain, in 2007.

**Methods:**

A cross-sectional study was conducted in a health-area setting of the Madrid Autonomous Region (*Comunidad Autónoma*), covering a population of 198,670 individuals aged over 14 years. Multiple correspondences were analyzed to identify the clustering patterns of the conditions targeted.

**Results:**

Forty-two percent (95% confidence interval [CI]: 41.8–42.2) of the registered population had at least one chronic condition. In all, 24.5% (95% CI: 24.3–24.6) of the population presented with multimorbidity.

In the correspondence analysis, 98.3% of the total information was accounted for by three dimensions. The following four, age- and sex-related comorbidity patterns were identified: pattern B, showing a high comorbidity rate; pattern C, showing a low comorbidity rate; and two patterns, A and D, showing intermediate comorbidity rates.

**Conclusions:**

Four comorbidity patterns could be identified which grouped diseases as follows: one showing diseases with a high comorbidity burden; one showing diseases with a low comorbidity burden; and two showing diseases with an intermediate comorbidity burden.

## Introduction

Over the course of the past century, improvements in living conditions and healthcare efficacy have led to an increase in the prevalence of chronic diseases. Furthermore, the prolongation of life expectancy means that the concurrence of more than one disease in any given individual is becoming increasingly frequent [1]–[5], a phenomenon defined as comorbidity or multimorbidity, depending on whether one is considering an association of diseases in relation to an index disease, or simply a relationship among multiple diseases without any one disease being taken as the point of reference.

Multimorbidity is present in one third of the adult population and its prevalence increases with age, reaching a prevalence of 60% among individuals aged 55 to 74 years [6]. Moreover, the existence of a tendency of some chronic diseases to form clusters has also been demonstrated [7].

When patients visit the physician, they tend to seek medical attention for more than one health problem. At each session, general practitioners (GPs) deal with an average of over three problems [8]. Individuals with multimorbidity register a higher mortality rate [9], occasion higher healthcare costs, and have: a higher risk of hospital admissions which, as ambulatory care sensitive conditions (ACSC), would otherwise be preventable [5]; a poorer perception of their physical and mental health; a poorer quality of life [10]; and a diminished functional capacity [11]. This is a challenge for GPs, who are tasked with treating patients rather than specific diseases. The result is that the whole is greater than the sum of the parts and [12], in view of its incidence and socioeconomic impact, multimorbidity thus constitutes a challenge to healthcare services in the 21^st^ century [13].

Research targets single diseases. Models of care for chronically ill patients are directed toward the management of each disease separately (disease management programs) and the clinical practice guidelines upon which such models are based also focus on single diseases [14].

Research into multimorbidity, which began relatively recently, is limited and under 3% of all published studies involve the primary care setting [6]. These studies lack uniformity in the definition of the concept of multimorbidity, type and number of diseases or conditions studied, data-sources used, and methods employed. This, together with the low number of studies, renders inter-study comparison difficult, and an international agenda for research in this field has thus been promoted [15] for the purpose of gaining in-depth knowledge of the characteristics of multimorbidity and adapting the care model to this new reality.

Most available studies focus on ascertaining prevalence and its distribution by age and sex [16]. Recently, some studies have been published which analyze the formation of chronic disease clusters [17], [18] but more remains to be learned about the clustering of chronic diseases and the risk factors that determine such clustering.

Accordingly, this study sought to identify comorbidity patterns in patients with chronic diseases, by reference to number of comorbidities, age and sex, in a population receiving medical care from 129 GPs in Spain, in 2007.

## Methods

We conducted a study in a health area setting in the Madrid Autonomous Region (*Comunidad Autónoma*), Spain, having a catchment population of 887,134, representative of the Spanish population in terms of age and sex.

Spain has a public healthcare system affording universal coverage, and all citizens are required to register with a physician: GPs provide care to the population over the age of 14 years, and subjects under this age receive pediatric care.

The study was based on the electronic medical records (EMRs) of 198,670 individuals, corresponding to the population registered with 129 GPs who, by way of inclusion criteria, met the following two EMR quality requirements: 1) they had kept notes on more than 64% of all visits received (75^th^ percentile); and, 2) they had recorded a mean of over four care episodes per patient across the study period (2007).

Data collected on patients included their respective ages, sex and all diagnoses for which they had visited the doctor in 2007. Patients were classified using the Adjusted Clinical Groups® (ACG®) Case-Mix System, version 7 [19]. ACGs are mutually exclusive groups of patients who make similar use of available resources over the course of the year (iso-resource groups). This classification system also generates categories termed “Expanded Diagnosis Clusters” (EDCs), which group patients on the basis of clinical criteria.

On the basis of pre-established criteria [20], the research team made an initial selection of 40 chronic EDCs, of which, taking prevalence and/or impact on health services into account, they finally selected 26.

All statistical analyses were performed using the SAS/STAT software package, and the graphs were designed with Stata 10. Qualitative variables were described by means of frequency distributions, along with their 95% confidence intervals where appropriate; in the case of quantitative variables, means and standard deviations were calculated. We calculated the prevalence of multimorbidity (two or more chronic diseases) and its 95% confidence interval and, taking each of the 26 chronic diseases as the index disease, described the comorbidity pattern vis-à-vis the other chronic diseases and quantified the proportion of patients corresponding to each of the six comorbidity levels.

Multiple correspondence analysis was used to obtain an overall idea of the data and the interrelationships among the different chronic diseases, taking age and sex into account. The method affords a graphic technique that displays each category as a point in a type of scatter plot. The positions of the category-points on this map indicate similarity or association between categories. Response-option points at the far end of the scatter plot show levels of repulsion between categories.

The method used followed the following steps: first, each chronic disease was dichotomized into 2 modalities (presence, absence), with each modality then being deemed a separate variable in the analysis. Age was classified into 7 categories. Finally a total of 28 variables with 61 different categories were included in the analysis. This meant that all the cross-tabulations of the 28 variables, including the cross-tabulations of the variables with themselves, were completely explained by 33 dimensions, i.e., the set of all the dimensions constitutes 100% of the original information. The existence of an association among the various categories allows for a reduction in the number of dimensions needed to explain the data, with some dimensions capturing more information than others. The amount of information explained by each dimension is evaluated using Benzécri inertia adjustment [21].

Second, several statistical parameters were calculated to characterize each dimension, namely: absolute contributions (partial contributions to inertia for each category); relative contributions (squared cosines for each category); and the category's position on the axis of the dimension. For any given dimension, the absolute contribution quantifies the importance of each category in that dimension. By knowing that the sum of the absolute contribution of the 61 categories in a given dimension is 1, one knows which category or categories are most important in the dimension in question. The importance of various categories in any given dimension explains the association among them. In our case, values above 0.05 were deemed important. For any given category, the relative contribution indicates its relevance to each of the dimensions. The sum of the value obtained for such a category's relative contribution in the 33 dimensions is equal to 1, thereby rendering it possible to ascertain in which dimension a category is most relevant, with its importance being increased if it is one of the dimensions to be explained. Lastly, a category's position in a dimension indicates association with categories having the same sign, and repulsion of or inverse relation with categories having the opposite sign.

Lastly, the category-points are plotted on different planes formed by the main dimensions, with the above-described statistical parameters being subsequently used to confirm or discard visual interpretations of the data. Clinical examination of this set of categories enables the dimension to be medically interpreted. The number of comorbidities is used as a supplementary variable in the correspondence analysis.

Ethical approval for this study was obtained from the Puerta de Hierro University Hospital Ethics Committee for Clinical Research (Record No. 252, dated February 22, 2010). Spain's 1999 Personal Data Protection Act (*Ley Orgánica de Protección de Datos de Carácter Personal*) requires that all patient registration data be treated confidentially. The data were organizad in a database and made available for analysis in this project and for research in future projects. The above Ethics Committee deemed the approach to be correct from a methodological and ethical standpoint and, in view of the fact that electronic records were used, waived the need for informed consent.

## Results

The 129 physicians participating in the study provided care to a registered population of 198,670 patients, mean age 43.2±18.5 years, comprising 104,003 women (52.3%), mean age 44.5±19.5 years, and 94,667 men (47.6%), mean age 41.8±17.3 years. In 2007, they saw a total of 149,409 patients, 75.2% of the registered population, mean age 45.6±19.6 years, made up of 84,704 women (56.6%), mean age 46.3±20.0 years, and 64,705 men (43.3%), mean age 44.7±18.9 years. In all, 42% of the registered population, 83,441 patients, mean age 54.1±19.5 years, received medical care for at least one of the 26 selected EDCs: the breakdown showed 50,126 women (60%), mean age 54.3±19.7 years, and 33,309 men (39.9%), mean age 53.8±19.2 years.

Of the above population, 42% (95% CI 41.8–42.2) had at least one chronic condition. Prevalence of multimorbidity (two or more conditions) was 24.5% (95% CI 24.3–24.6), and was higher among women (28.1±0.1) than men (19.4±0.1), a difference of 8.7 points (95% CI 8.3–9.0). Prevalence increased progressively with age until 69 years, and tended to stabilize thereafter.

The correspondence analysis enabled 98.3% of total inertia to be explained by three dimensions, with the first accounting for 82.4%, the second 9.3% and the third the remaining 6.6%.

**Figure 1:**
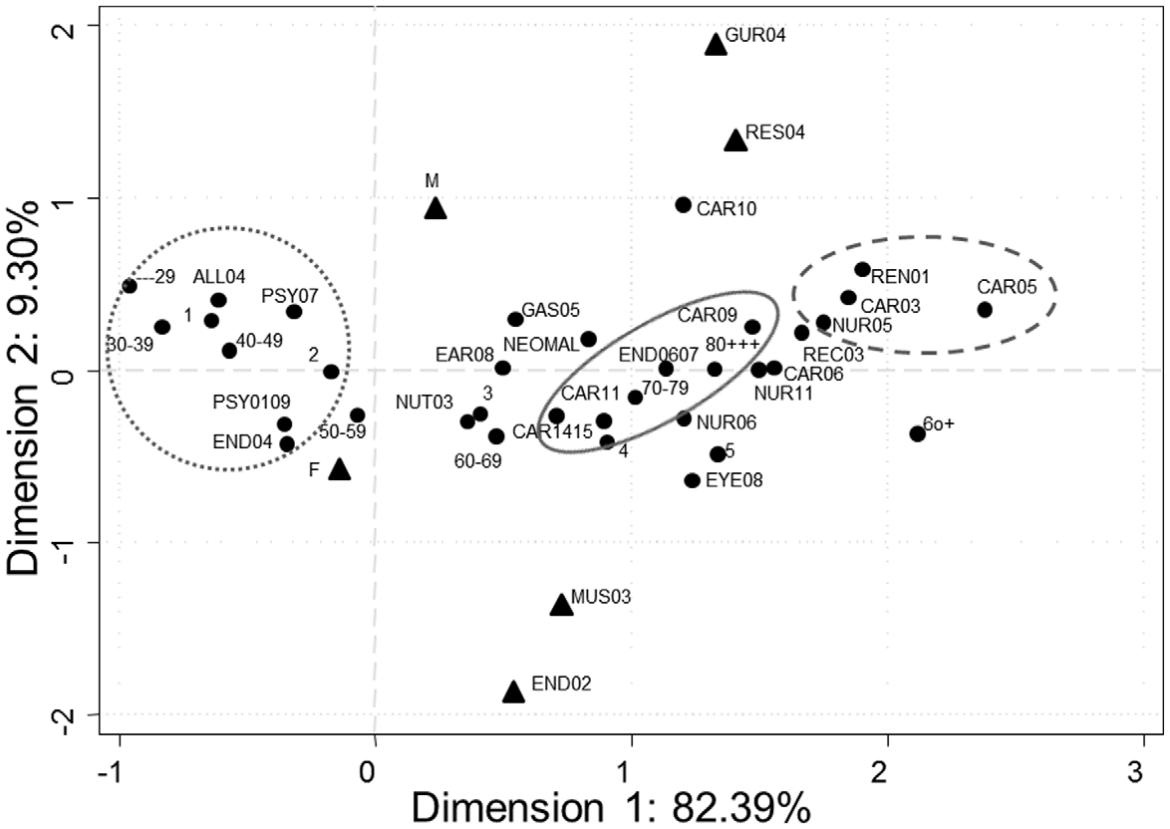
Graphical depiction of chronic diseases, age and sex. Correspondence analysis showing the projections on the plane defined by dimensions 1 and 2. M: Male, F: Female. ▴Variables most representative of dimension 2. ______ Pattern A. ---------- Pattern B. ………. Pattern C.

**Figure 2:**
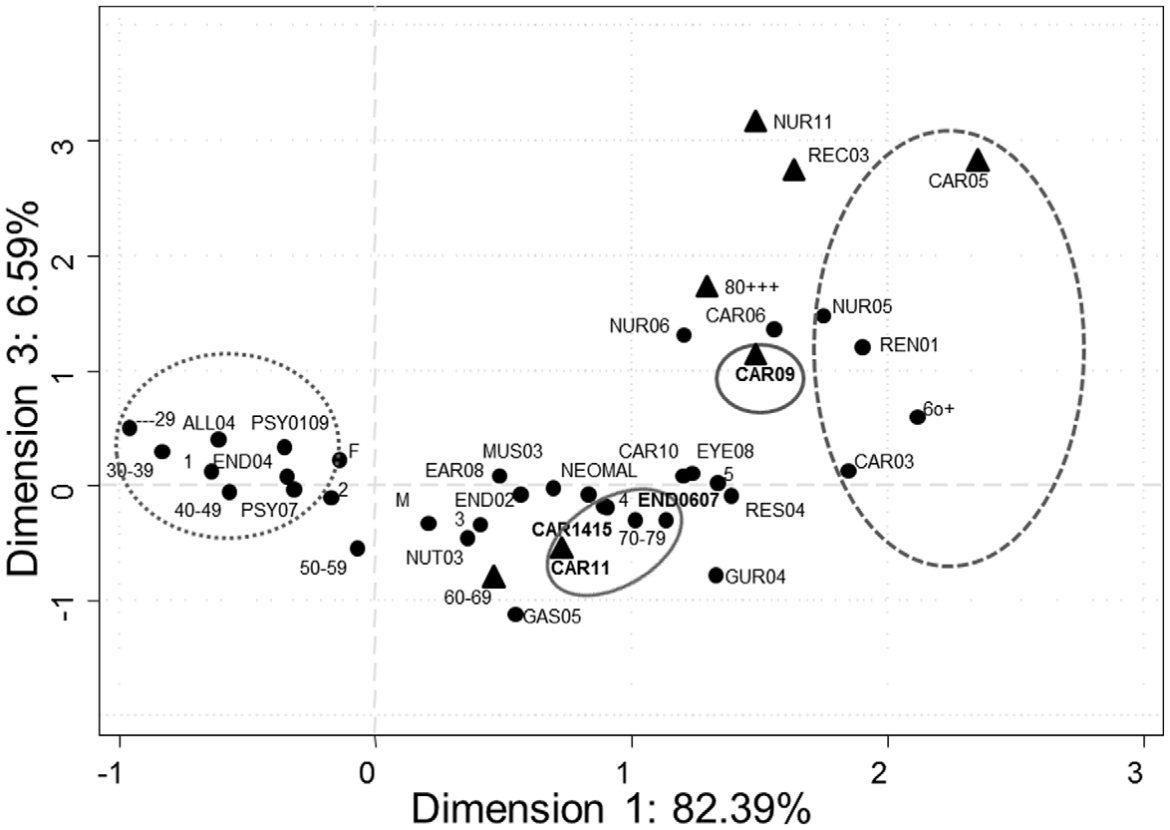
Graphical depiction of chronic diseases, age and sex. Correspondence analysis showing the projections on the plane defined by dimensions 1 and 3. M: Male, F: Female. ▴ Variables most representative of dimension 3. ______ Pattern A. ---------- Pattern B. ………. Pattern C.

Figures 1 and 2 depict the relationships among the multiple categories of the variables, the position of each on the axes of the first and second dimensions, and the positions of these diseases in the third vis-à-vis the first dimension.

The first dimension showed a group of diseases (pattern A) which, in addition to explaining a substantial part of the information corresponding to this dimension (absolute contribution values of over 0.05), strongly represented this dimension (high relative contribution values). On the axis of the first dimension (Figure 1), these diseases occupied positions corresponding to intermediate positive values, ranging from 0.71 to 1.47. This group included cardiac arrhythmias (CAR09), hyperlipidemia (CAR11), hypertension with and without complications (CAR1415), and diabetes with and without complications (END0607), diseases which, without exception, are strongly attracted by the patient stratum aged 70 years or older.

The second group of diseases (pattern B) making up the first dimension (Figure 1) included ischemic heart disease (CAR03), cerebrovascular diseases (NUR05), chronic renal failure (REN01), and congestive heart failure (CAR05), all of which are attracted by patients over 80 years of age, and repelled by the stratum of patients with ages under 40 years. In this second group, the diseases on the axis of the dimension occupied positions corresponding to high positive values, ranging from 1.75 to 2.38, in addition to having a certain amount of inertia captured from the first dimension, which was lower than that of the first group of diseases but higher than 0.03. This second group of diseases occupied positions similar to those of categories 5 and 6 or more comorbidities than the supplementary variable, and there was thus a strong attraction among them.

The third group of diseases (pattern C), associated with the stratum of patients aged under 30 years and, in turn, attracted by the category of the supplementary variable of one associated comorbidity (Figure 1), were: asthma (ALL04); thyroid disease (END04); anxiety or depression (PSY0109); and schizophrenia (PSY07). This third group of diseases did not characterize the first dimension, since its contribution to the inertia of the dimension was limited and the values of its relative contribution were close to zero. This group of diseases occupied negative positions on the axis of the first dimension, similar to those of categories, “1 or more” and “2 or more” comorbidities.

The remaining diseases made up the fourth group (pattern D), with positions similar to those of the first group of diseases and poorly represented by the first dimension.

Table 1 shows the case-frequency distribution for each condition at each level of comorbidity, grouped according to the different comorbidity patterns identified in the correspondence analysis. While some conditions, such as asthma, schizophrenia, anxiety/depression and thyroid disease, registered a low morbidity burden, others such as heart failure, ischemic heart disease, cerebrovascular disease and chronic renal failure, registered a high comorbidity burden. Finally, a larger set, comprising groups B and C, displayed an intermediate comorbidity burden.

**Table 1:** Comorbidity associated with chronic diseases.

EDC	No. Cases	ID only	ID+1 or more	ID+2 or more	ID+3 or more	ID+4 or more	ID+5 or more
**PATTERN A**							
Hypertension (with and without complications) [CAR1415]	28760	11.05	88.95	67.95	44.05	24.45	12.10
Disorders of lipid metabolism [CAR11]	22345	13.19	86.81	65.76	43.29	24.58	12.49
Type 2 diabetes (with and without complications) [END0607]	10058	9.67	90.33	74.55	54.11	33.86	18.86
Cardiac arrhythmia [CAR09]	5777	8.60	91.40	78.43	61.28	42.08	25.36
**PATTERN B**							
Cerebrovascular disease [NUR05]	2658	5.91	94.09	83.07	66.59	46.46	28.33
Ischemic heart disease (excluding AMI) [CAR03]	2344	3.97	96.03	86.90	70.56	49.40	32.81
Chronic renal failure [REN01]	1964	5.86	94.14	84.57	69.25	51.12	33.50
Congestive heart failure [CAR05]	1377	3.05	96.95	90.12	78.29	61.26	42.19
**PATTERN C**							
Anxiety and depression [PSY0109]	27357	36.46	63.54	39.54	24.38	13.91	7.14
Thyroid disease [END04]	19299	31.97	68.03	42.49	26.26	15.12	7.86
Asthma [ALL04]	7614	40.74	59.26	34.98	21.55	13.12	7.17
Schizophrenia and affective psychoses [PSY07]	1309	31.02	68.98	44.16	24.68	13.67	7.87
**PATTERN D**							
Obesity [NUT03]	19640	17.11	82.89	60.58	39.76	22.68	11.36
Osteoporosis [END02]	6143	9.56	90.44	72.54	49.44	29.01	14.76
Deafness, hearing loss [EAR08]	5403	18.88	81.12	61.08	42.88	27.63	15.57
Malignant neoplasms [NEOMAL]	5138	14.25	85.75	66.62	46.44	28.67	15.84
Degenerative joint disease [MUS03]	4452	11.25	88.75	72.24	52.34	32.79	17.65
Benign prostatic hypertrophy [GUR04]	4089	11.49	88.51	68.38	45.98	27.42	14.97
Emphysema, chronic bronchitis, COPD [RES04]	3183	9.80	90.20	72.86	54.16	35.38	21.08
Generalized atherosclerosis [CAR10]	2705	13.20	86.80	72.16	55.75	39.26	23.51
Glaucoma [EYE08]	2450	7.31	92.69	79.47	60.98	40.49	23.80
Chronic liver disease [GAS05]	2121	13.11	86.89	67.61	48.33	32.81	18.39
Dementia and delusions [NUR11]	1112	11.33	88.67	73.38	52.25	33.45	19.06
Chronic skin ulcer [REC03]	955	8.90	91.10	77.49	58.95	41.05	24.91
Cardiac valve disease [CAR06]	936	7.69	92.31	82.16	64.10	47.54	30.02
Parkinson’s disease [NUR06]	805	9.57	90.43	77.14	55.53	38.63	26.09

EDC: expanded diagnosis cluster; ID: index disease; COPD: chronic obstructive pulmonary disease; AMI: acute myocardial infarction.

The second dimension (Figure 1) was made up of two sex-related disease groups, namely, a first group that included osteoporosis (END02) and degenerative joint disease (MUS03), associated with the female sex, and a second group, comprising benign prostatic hyperplasia (GUR04) and chronic obstructive pulmonary disease (COPD) (RES04), associated with the male sex. In addition to being well represented by the dimension, these diseases had a high relative contribution value compared with that for the remaining dimensions, contributing to the inertia of the second dimension with values of over 0.05. Their positions on the axis of the second dimension (negative or positive positions, with extreme values in both cases) were sex-related.

The third dimension (Figure 2) explained the presence of the following diseases in patients over the age of 80 years: congestive heart disease (CAR05); cardiac arrhythmia (CAR09); dementia (NUR11); and chronic ulcer (REC03). This group of diseases, associated with the age stratum of 80 years and older, was repelled by lipid metabolism disorders CAR 11, a disease associated with patients aged 60 to 70 years.

## Discussion

Our study shows that 42% of the population visiting the GP's office has at least one chronic condition, and that close on a quarter of such subjects have two or more of these diseases. Four comorbidity prevalence patterns were identified, namely, one showing high, one showing low, and two showing intermediate comorbidity rates.

Comparing our results to those of studies published to date proves difficult, however, because the latter not only address different index and associated diseases, but some are based on patient-reported data, some on administrative databases, and others on medical records.

Our study analyzed the association among 26 chronic health conditions. As other studies, ours included diseases and risk factors, both to facilitate comparison and by reason of their relevance in terms of healthcare resource use.

The greatest limitation of our study lies in the data-source used. We relied on data on the population over 14 years of age who received medical attention at their GP's office, using the diagnoses shown in the EMRs to compute the number of cases. As a data-source for morbidity studies, medical histories introduce biases stemming from the completeness and quality of the record kept [22]. To minimize this problem, physicians were selected who offered the greatest assurance of quality in their records. Despite such limitations, the prevalence of multimorbidity as estimated by GPs' medical records is substantially higher than that reported by general population surveys [23], and similar to that found in population-based longitudinal studies [24].

Another study limitation derives from the diseases selected. We chose those that are frequently seen in general practice or represent a considerable health-service burden, since we regard these as being the most relevant. Nonetheless, this criterion may have introduced a bias, thereby increasing the frequency of comorbidity and generating groups that might have been different, had we included diseases that were less frequent or registered less health service impact.

The prevalence of comorbidity is determined by the number of associated conditions studied [23], [25]. Our study, based on 26 chronic conditions, detected a crude prevalence of multimorbidity of 24.5% in the population over 14 years of age, which was higher in women than in men and increased with age. Comparing our findings to those of other GP record-based studies shows that: in Australia, Britt et al. [26], using a number of chronic conditions similar to ours, with no age restrictions, reported a multimorbidity rate of 29%; in The Netherlands, the authors of a study with no restrictions on age and an open list that included acute and chronic processes, observed a multimorbidity rate of 29.7% [25]; and in Spain, a population-based study that included a comprehensive list of chronic diseases estimated a multimorbidity prevalence rate of 30% [27].

Correspondence analysis is an exploratory, multivariate technique that converts a data matrix into a type of scatter plot, in which the rows and columns are depicted as points. Though widely known, this method is nevertheless rarely used to analyze multimorbidity data [28]. Other research into multimorbidity patterns has used cluster analysis to identify morbidity patterns [17]. This type of analysis assigns each disease to only one cluster, a rather unrealistic approach in that some diseases can be expected to be part of more than one pattern. As a result, recent research has turned to factor analysis [18], whereby dichotomous diagnoses are transformed as continuous variables. Our decision to use correspondence analysis was based on the fact that it is really a principal components analysis of categorical data and is a multivariate method which enables one to obtain an overall idea of the data and the interrelationships among the various diseases. The chronically ill patients in our study registered four patterns of comorbidity but, in every instance, over half the cases were associated with at least one other chronic condition. Other studies have also established different comorbidity profiles by reference to an index disease [26], [29], and have demonstrated a higher rate of comorbidity in patients with heart and cerebrovascular diseases, and a lower rate in patients with asthma and mental disorders. A recent study addressing chronic respiratory diseases [30] reported different patterns for asthma and COPD. In an earlier study on heart failure, we found that these patients have a very high comorbidity burden [31].

Based on the chronic diseases chosen, comorbidity was the rule in our study, and only one small chronic disease group (pattern C) displayed a low comorbidity burden. In such circumstances, health service coordination and integration becomes a necessity. Disease management programs, targeting a specific illness, have limited application in the care of chronic patients and are restricted to those included in the low comorbidity pattern. Case management programs would appear to offer a more logical alternative, with GPs, by virtue of their involvement in the care of the index disease and other conditions, having to assume a pivotal role as case managers in respect of such patients [29].

Comorbidity has an impact on health outcomes [32]. Although clinical research stresses the internal validity of clinical trials, aspects linked to external validity tend to be overlooked. Consequently, the feasibility of extrapolating the research findings to clinical practice is limited [33], [34]. Clinical decisions should take relevant clinical trials into account, and studies that produce results which are applicable to routine clinical practice are relevant [35]. All in all, relevance depends on external validity, and in this respect, comorbidity is a key factor which is, nevertheless, only taken into account in clinical trials for the purpose of excluding patients affected by it.

Furthermore, clinical practice guidelines and disease management programs usually focus on specific diseases and fail to take the presence of comorbidity into consideration. In the light of our results, there is a small group of chronic conditions (pattern C) which tend to be isolated entities. The majority of the chronic conditions are, however, associated with one another. This is especially true of one group (pattern B), in which it is interesting to note that the diseases involved very rarely develop as isolated entities and are most frequently associated with a high comorbidity rate. In such cases, the application of the results of research studies is questionable, and there is thus a clear need for new clinical practice guidelines which take into account the comorbidity of patients presenting with any disease in this group.

Comorbidity limits the capacity for self-care [36]. Integrated care for the problems of the chronically ill patient with comorbidity improves overall outcomes and adherence to treatment [37], and continuity serves to reduce healthcare costs [38]. In such a context, a longitudinal, generalist approach may be the most suitable strategy [39] and, given their generalist training, family physicians need to play a central role in both the care and coordination of care of chronically ill patients.

In conclusion, the correspondence analysis enabled us to identify four comorbidity patterns that grouped diseases as follows: one showing diseases with a high comorbidity burden; one showing diseases with a low comorbidity burden; and two showing diseases with an intermediate comorbidity burden.

Identification of these comorbidity patterns in patients with chronic diseases gives rise to new questions, such as: is the same healthcare model valid for all four groups?; once a given disease has been identified, could the diagnosis of another in the same group be envisaged?; why do only some and not all diseases tend to occur in association with others?; and, what characteristics or risk factors are shared by diseases which tend to occur in association with others?
